# The role of androgens in follicle maturation and ovulation induction: friend or foe of infertility treatment?

**DOI:** 10.1186/1477-7827-9-116

**Published:** 2011-08-17

**Authors:** Norbert Gleicher, Andrea Weghofer, David H Barad

**Affiliations:** 1 Center for Human Reproduction - New York, New York, USA; 2 Foundation for Reproductive Medicine, New York, New York, USA; 3 Department of Obstetrics, Gynecology and Reproductive Sciences, Yale University School of Medicine, New Haven, Connecticut, USA; 4 Department of Obstetrics and Gynecology, Medical University Vienna, Vienna, Austria; 5 Departments of Epidemiology and Social Medicine and Obstetrics, Gynecology and Women's Health, Albert Einstein College of Medicine, Bronx, New York, USA

## Abstract

**Background:**

Effects of androgens on follicle maturation have been controversial for some time. Here, we review the potential of their applications in improving human ovulation induction, based on human and animal data, reported in the literature.

**Methods:**

We reviewed the published literature for the years 2005-2011, using relevant key words, in PubMed, Medline and Cochrane reviews, and then performed secondary reviews of referenced articles, which previously had not been known or preceded the searched time period. A total of 217 publications were reviewed.

**Results:**

Contrary to widely held opinion, recent data, mostly developed in the mouse, convincingly demonstrate essential contribution of androgens to normal follicle maturation and, therefore, female fertility. Androgens appear most engaged at preantral and antral stages, primarily affect granulosa cells, and exert effects via androgen receptors (AR) through transcriptional regulation but also in non-genomic ways, with ligand-activated AR modulating follicle stimulating hormone (FSH) activity in granulosa cells. While some androgens, like testosterone (T) and dehydroepiandrosterone (DHEA), appear effective in improving functional ovarian reserve (FOR) in women with diminished ovarian reserve (DOR), others may even exert opposite effects. Such differences in androgens may, at least partially, reflect different levels of agonism to AR.

**Discussion:**

Selective androgens appear capable of improving early stages of folliculogenesis. They, therefore, may represent forerunners of a completely new class of ovulation-inducing medications, which, in contrast to gonadotropins, affect follicle maturation at much earlier stages.

## Background

Understanding of effects of androgens on follicle maturation and ovulation induction has recently undergone considerable change. Primarily because of reported negative effects in mouse models [1-4], androgens, for the longest time, almost universally were considered detrimental to normal folliculogenesis. Poor oocyte quality observed in human hyperandrogenic polycystic ovary syndrome (PCOS) contributed to this opinion [5]. Some recent mouse studies also still emphasize adverse androgen effects on oocyte meiotic capacity above certain concentrations [6].

The picture now evolving, based on androgen receptor (AR) studies in the mouse [7,8], is, however, more complex and potentially reflects substantial theoretical and practical relevance to therapeutic ovulation induction in humans. We, here, therefore, present a literature review on the subject, primarily concentrating on developments after 2007, the cut off date of a review on the subject by Laufer, published in 2009 [9].

### Literature search strategy

We performed a literature search of PubMed, Medline and Cochrane reviews for the years 2005-2011 under the following key phrases/words: < androgens/testosterone (T)/androstenedione (ASD)/dehydroepiandrosterone (DHEA)/aromatase inhibitors (AI) in spontaneous ovulation>, < androgens/T/ASD/DHEA/AI in ovulation induction>, < androgens/T/ASD/DHEA/AI in folliculogenesis>, < androgens/T/ASD/DHEA/AI in follicle maturation>, < androgens/T/ASD/DHEA/AI and meiosis>, < androgens/T/ASD/DHEA/AI and aneuploidy>, < androgens/T/ASD/DHEA/AI in infertility> < androgens/T/ASD/DHEA/AI in in vitro fertilization (IVF)>, < androgens/T/ASD/DHEA/AI in diminished ovarian reserve (DOR)>, < androgens/T/ASD/DHEA/AI and luteinizing hormone (LH)>, < androgens/T/ASD/DHEA/AI and follicle stimulating hormone (FSH)>, < androgen receptors (AR) in folliculogenesis/follicle maturation/oocyte maturation>.

Relevant manuscripts were identified and their reference lists reviewed for additional references, which either were not discovered through above described literature search or preceded the search period. Combined, this resulted in an initial review of 217 publications. Since the review by Laufer, published in 2009, covered the subject well up to 2007 (58/60 references were pre-2007) [9], we concentrated on manuscripts published after 2007, listing here 101 references.

### Animal models

The most profound changes in understanding effects of androgens on folliculogenesis come from *in vivo *animal data, *in vitro *follicle culture bioassays, and studies involving the AR. Androgens primarily exert effects through transcriptional regulation by the nuclear AR [10]. Increasing evidence, however, now also points towards non-genomic androgen signaling [7,11,12].

### Effects of androgens on the ovary

As summarized by Lenie and Smitz [10], androgens at sub-nanomolar concentrations exert genomic and non-genomic effects. As a ligand-activated transcription factor, AR detects sub-nanomolar androgens in cytoplasm, and converts androgen signals into changes in gene expression [13]. Rapid non-genomic signaling of androgens, ultimately, may modulate transcriptional activities of AR. They also note that, amongst the various ovarian cell types, most AR expression can be found in granulosa cells.

**Figure 1:**
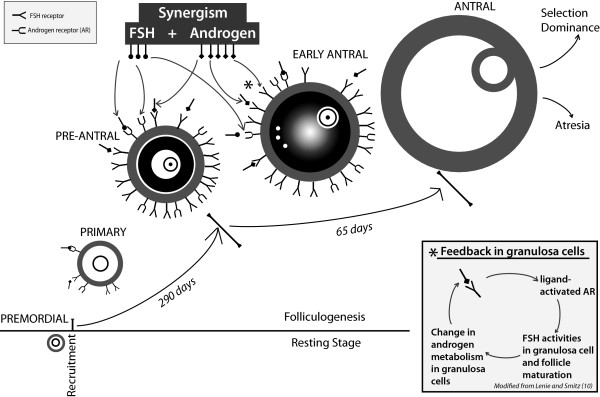
**Synergism between androgen and FSH**. The figure depicts the potential synergism of androgens and follicle stimulating hormone (FSH) during early folliculogenesis. Here in detail depicted only on pre-antral and early antral follicles, the figure is meant to demonstrate the high concentration of androgen receptor (AR) at pre-antral to antral stages, declining thereafter [10,15-17]. High concentrations of AR at these stages are strongly suggestive of peak androgen effects at these stages of folliculogenesis. Androgens primarily affect granulosa cells [21] through transcriptional regulation via AR but do so also via non-genomic ways, with ligand-activated AR modulating FSH activity in granulosa cells. The box in the right lower quadrant schematically demonstrates the synergism between androgens and FSH, based on Lenie and Smitz [10], practically creating a feed back loop.Synergism between androgens and FSH suggests the possibility of new pharmacologic approaches to ovulation induction, utilizing this synergism in early folliculogegesis to improve oocyte numbesr and quality. For further detail, see text.

Androgens affect follicle maturation from very early stages on: In bovine follicles Yang and Fortune reported T to stimulate transition from primary to secondary follicle [14]. Relevant to the clinical human experience, in rodents and primates *Ar *mRNA and AR protein appear in highest concentrations in ovaries containing mostly immature preantral and early antral follicles. *Ar *mRNA and AR protein then decrease with advancing follicle maturation [15-17], suggesting a primary importance for androgens especially in early stages of follicle maturation (Figure 1).

Preantral and early antral follicles constitute a majority of what we in clinical practice have come to call functional ovarian reserve (FOR), best assessed by anti-Müllerian hormone (AMH) and antral follicle count (AFC) [18]. Positive and/or negative effects of androgens should, therefore, be reflected in AMH levels and AFCs (see also later).

Not all androgens are equally agonistic for the AR. T and its metabolite 5α-dihydrotestosterone (DHT) are the most potent naturally occurring agonists [10]. Ovaries and adrenals produce three distinct androgens during steroidogenesis: ASD, T and DHEA. Since their agonism with AR differs, varying effects of androgens appear likely (see also later). Moreover, one also has to consider the possibility that metabolites of these androgens exert effects, possibly via different mechanisms and/or receptors. Estrogenic effects via estrogen receptors are a possible example.

Lutz et al reported that only T and ASD potently induced oocyte maturation in *Xenopus*, whereas DHT actually inhibited T- and human chorionic gonadotropin (hCG)-induced maturation and signaling [11]. Differences in reported clinical androgen effects may, therefore, also be consequence of varying agonistic AR effectsby different androgens.

Whichever androgens induce ligand-activated AR, all potentially modulate FSH activities in developing granulosa cells. They, therefore, serve as important modulators of granulosa cell differentiation and follicle maturation, especially during particularly FSH-dependent, early antral stages [10]. As already noted earlier, androgenic effects on follicle maturation, therefore, appear especially pronounced at these early stages of folliculogenesis (Figure 1).

Interactions between ligand-activated AR and FSH can, thus, be viewed as a form of synergistic "feed back" since granulosa cells respond to FSH at those stages of development with changes in androgen metabolism. This happens due to changes in expression in steroidogenic enzymes P450scc, P450arom, and 5α-reductase, in turn determining type of androgen and relative distribution of the three main androgens in follicles [10]. These androgens, in turn, as noted previously, can affect the developing follicle in genomic (AR) and non-genomic ways (diffusion, etc). Clinically, one, therefore, would expect synergistic effects of androgens and FSH at these stages of follicle maturation (to be further discussed later, and Figure 1).

Lenie and Smitz demonstrated in a mouse *in vitro *folliculogenesis model the importance of AR to normal follicle maturation. By treating follicles with anti-androgenic compounds, follicle growth during the preantral phase was reduced, the steroidogenic environment was altered, and, probably most importantly, oocyte meiotic maturation in response to human hCG was arrested [10]. Androgen receptor knock out (ARKO) models provide further clarity.

### ARKO mouse models

Walters et al recently summarized how ARKO mouse models are able to decipher AR-mediated female reproductive functions [19]. AR is expressed in various reproductive tissues, including, as noted, in different ovarian cells and neuroendocrine tissues, like pituitary and hypothalamus [20]. Sen and Hammes recently elegantly elucidated AR effects in different organs/tissues by generating granulosa cell- and oocyte-specific ARKO mice, allowing determination of where androgen-dependent effects were located [21]: Almost all reproductive phenotypes observed in global ARKO mice proved explainable by AR expressions in granulosa cells, including premature ovarian failure (POF), subfertility with longer estrous cycles and fewer ovulated oocytes, more preantral and atretic follicles, fewer antral follicles and fewer corpora lutea. In addition, *in vitro *growth of follicles was slower than in control wild-type animals.

Oocyte-specific ARKO mice, in contrast, demonstrated none of these phenotypes, and androgens no longer promoted oocyte maturation. ARKO mice, due to disruptions in AR signaling, also exhibited altered uterine development, though not reduced fertility [21]. Fertility impairment in ARKO mice appears, thus, primarily ovarian in nature.

Oocytes, however, also appear to benefit from androgen effects. Li et al reported that T positively contributes to porcine oocyte meiotic resumption. AR apparently significantly contributes to T-induced mitogen-activated protein kinase activation and germinal vesicle breakdown [22].

### Androgen production and therapeutic ranges

Theca cells provide all androgens required by developing follicles. They then are converted by granulosa cells through the action of P450 aromatase into the estrogens, estrone and 17β-estradiol [23]. Via aromatization, androgens may, thus, also exert effects through the estrogen receptor.

In gonadotropin-dependent stages of folliculogenesis, androgen production is controlled by luteinizing hormone (LH) [24]. *In vitro *culture of theca cells also results in androgen production with low level LH stimulation; but under high level LH, ASD production is inhibited and progesterone secretion is stimulated, suggesting luteinization of theca cells [25].

Applying this clinically, Shoham suggested a "therapeutic window" range for LH [26], and we proposed relative therapeutic ranges for all hormones, based on in-range agonist/antagonist ratios rather than specific hormone levels [27]. Romero and Smitz recently, indeed, demonstrated a therapeutic range for androgens in mouse ovarian follicles [6].

In an *in vitro *follicle bioassay they investigated exogenous addition of ASD and T. Both androgens altered endogenous production of ASD, T, estradiol and progesterone, and affected oocyte capacity to resume meiosis. ASD at concentrations above 200 nM induced increased T and estradiol production. Exposure to T above 200 nM induced, however, elevated levels of estradiol and progesterone, while exposure to ASD, as well as T, at those concentrations demonstrated dose-dependent negative effects on polar body extrusion. ASD-treated follicles, in addition, also produced oocytes with chromosome displacements on the metaphase plate, while follicles exposed to 25 mIU/mL FSH, 3 mIU/mL hCG and elevated aromatizable androgens altered steroid profiles, and demonstrated abnormal follicle development as well as impaired oocyte meiotic competence.

Using rat granulosa cells, Wu et al recently demonstrated that T in absence of gonadotropins increases aromatase (Cyp19) and P450scc side-change cleavage expression, both enzymes of major importance for normal ovarian function. Cyp19 converts T into estradiol, while 5α-reductase converts it into the pure androgen 5α- DHT [28]. They also demonstrated in this study that T directly affects the expression of genes involved in granulosa cell differentiation.

How sensitive the developing follicle is to fluctuations in hormone substrates was recently also demonstrated by Sánchez et al, who reported that FSH concentrations alter gene expression in the cumulus-oocyte complex in mice. Even as small as a 2.5-fold increase in FSH changed oocyte and cumulus cell transcript levels. Decreasing FSH levels did not change transcript levels but limited inappropriate luteinizing hormone/chorionic gonadotropin receptor (Lhcgr) expression [29], a finding potentially relevant to the discussion about clinical effects of higher versus lower gonadotropin dosages during ovulation induction.

Like other hormones, androgens, thus, apparently function within therapeutic ranges, but vary in their effects on follicle and oocyte even at similar concentrations. Both of these observations can, at least partially, explain the many contradictory reports of androgen effects in female infertility. Table 1 summarizes hormonal ovarian effects of androgens in various animal models.

**Table 1:** Summary of androgen effects on maturing follicles based on animal data

Observation	Potential clinical relevance
Different androgens affect ovarian follicles in varying ways	May explain positive and negative effects reported in literature; Different androgens can be expected to have different clinical effects.
Androgens will affect follicles differently at varying concentrations	Same as above
Androgens affect ovaries through genomic (AR) and non-genomic signaling	
• Most affected cell type: granulosa cells	Granulosa cell abnormalities should be associated with POF and other ovarian function abnormalities
• Most affected maturation stage: preantral and early antral follicles	Androgen effects should be visible in AMH levels and AFCs
Androgens activate FSH activity on granulosa cells the most at antral stage	Antral stage should be most responsive to synergistic activity of androgens and FSH

In addition to endocrine effects, androgens also exert immune effects with significance on ovarian function. Si et al reported in a mouse model that glucocorticoids and androgens equally ameliorate POF [30]. Since autoimmunity appears increased in infertile women [31], androgens may also beneficially affect ovarian function by serving as antagonists towards strong female autoimmune predisposition [32], which quite often is directed against the ovary [33].

### Human Data

As noted, androgens have mostly been considered clinically detrimental to follicle development. Aside from early animal data [1-4], this was mostly due to the hyperandrogenism observed in many classical PCOS patients, associated with anovulation and inferior oocyte quality in IVF [5]. PCOS, therefore, represents a good starting point for a review of human data.

### PCOS as a model of androgen excess

Qiao and Feng recently described hyperandrogenemia as one of the "intra-ovarian" factors characterizing PCOS and affecting oocyte maturation. They note that hyperandrogenemia is typically attributed to excessive androgen production by ovaries, with substantial contribution from adrenals and, to a lesser extent, from adipose tissue. High androgen levels in women with insulin resistance can also be due to inhibition of hepatic production of sex hormone-binding globulin (SHBG) [5].

Also according to these authors, studies suggest adverse effects of high androgen levels on oocyte developmental competence [34-36]. Typically, small PCOS follicles are hyperandrogenic due to raised androgen production by theca cells [37,38].

PCOS is a heterogeneous ovarian syndrome, characterized by a multitude of reproductive-endocrine as well as metabolic phenotypes [5]. One, however, also could argue that polycystic ovaries, themselves, represent an ovarian phenotype, shared by hyperandrogenic and normo-androgenic females. Since only some PCOS patients are hyperandrogenic, hyperandrogenemia cannot be the only etiology of PCOS [5].

Because of the heterogeneity of PCOS patients, it is difficult to interpret published outcome data after infertility treatments. Since large numbers of very small follicles (antral follicles of 2 - 5 mm size) define the diagnosis, it is not surprising that, independent of underlying etiology, all PCOS patients characteristically produce large oocyte yields after gonadotropin stimulation [39]. Some authors have suggested, though, that oocyte quality lags, resulting in lower implantation, and higher miscarriage rates as well as more aneuploidy [40,41]. Others reported no meaningful outcome differences between PCOS and control patients in IVF cycles [42,43]. Weghofer et al determined that, over all, PCOS patients produce larger euploid embryo numbers, yet still demonstrate lower pregnancy and higher miscarriage rates [44], suggestive of a non-chromosomal quality factor in oocytes.

Differences in reported outcomes most likely are a consequence of noted etiological heterogeneities. This is supported by our recent report of an ovarian PCOS phenotype, highly associated with a specific *FMR1 *sub-genotype (*het-norm/low*), neither associated with obesity nor hyperandrogenism but with rapidly depleting ovarian reserve. Affected women at young ages demonstrate classical PCOS but at mid- and older ages often exhibit abnormally diminished FOR [33]. The PCOS phenotype is, therefore, not stable presence in all PCOS patients, and can disappear.

Some animal data are relevant to a better understanding of PCOS in humans: For example, it has been known for some time that in animal models dehydroepiandrosteron (DHEA) can induce PCOS phenotypes in previously normal ovaries [45,46]. In humans we demonstrated the same only more recently, when long-term DHEA supplementation, in women with even very severe DOR was shown to result in typical PCOS-like ovaries [47] (for further detail, see below).

Increased T and/or LH levels in women with PCOS have been widely associated with elevated AMH levels, and have been suggested as causes of impaired follicle/oocyte development and embryo quality [38,48,49]. High serum AMH levels, however, do not necessarily indicate high intrafollicle AMH levels. We, for example, demonstrated that AMH per oocyte retrieved varies based on *FMR1 *genotypes and sub-genotypes [50]. Whether intrafollicle AMH levels reflect the quality of follicle maturation has, indeed, remained highly contested. Probably as many investigators concluded that high intrafollicle AMH in PCOS patients is harmful, as have reached the opposite conclusion [5].

Currently the only X-linked candidate gene for PCOS is the AR gene [51]. The gene's CAG repeat polymorphism has been reported associated with a PCOS phenotype [52]. As Van Nieuwerburgh et al recently reported, shorter CAG repeats in the AR gene appear to enhance androgenicity in PCOS. Specifically, PCOS patients with bi-allelic means of less than 21 repeats had lower DHT, ASD, LH levels, LH/FSH ratios and more clinical signs of hyperandrogenism, like acne and hirsutism. Most other authors were, however, unable to confirm such associations [53-55].

Such opposing conclusions may, once again, be a consequence of patient selection, reflective of different underlying PCOS pathophysiologies, and, perhaps, genotypes. The prevalence of polymorphisms may, however, also differ based on racial/ethnic backgrounds of study populations, as recently demonstrated with CGG repeat polymorphisms in the *FMR1 *gene [56]. Dasgupta et al, studying South Indian women, concluded that CAG repeat polymorphism, by itself, does not represent a useful marker of PCOS. They, however, noted among PCOS patients a trend towards preferential activation of shorter alleles (among individuals with non-random X-inactivation) [57].

Returning to animal models, a causal relationship between hyperandrogenism and PCOS is also suggested by Yang et al [58], who tried to elucidate the molecular mechanisms of excessive androgens in a mouse PCOS model. Culturing neonatal mouse ovaries with T, mainly containing primordial follicles, they demonstrated increases in primary follicles via the phosphatidylinositol 3-kinase/Akt pathway, and demonstrated further that androgens induced Forkhead box (Foxo)-3a activation, and translocation of Foxo3a protein from oocyte nuclei to the cytoplasm, likely a key step in the activation of primordial follicles. That T may, indeed, enhance follicle recruitment in PCOS, has previously already been suggested by Qureshi et al [59].

T was, however, also able to down-regulate the expression of growth and differentiation of factor-9 expression via its receptor, and, therefore, can in this mouse PCOS model induce arrest of follicle development [58]. T, thus, in this model appears able to improve recruitment of resting primordial follicles but also potentially contributes to arrest of developing follicles. Looking at T in its potential clinical utility, T, thus, once again, appears able to exert positive and negative effects on follicle maturation.

PCOS data from animal models, therefore, contribute two important facts of potential clinical relevance to humans: Androgens appear to enhance follicle recruitment, and, potentially, can have positive *as well as *negative effects on follicle maturation; not only, as still widely believed, negative ones.

Heterogeneity of PCOS phenotypes in humans, likely, is the principal reasons why these opposing effects, so far, have not been yet isolated, which in clinical practice would make them better targets for individualized therapeutic interventions.

### DHEA in human subfertility

Within such a context it is noteworthy that DHEA has found increasing clinical application in the treatment of diminished ovarian reserve, recently reviewed in detail elsewhere [60].

Once again briefly returning to the mouse, we previously noted that a DHEA-induced mouse model of PCOS has been utilized in a variety of research disciplines [45,46]. It was actually first reported in the rat by Lee et al [61], and only later applied to mouse experiments [45,46,62,63].

Belgorosky et al replicated some of the above-discussed T-induced observation with DHEA in the mouse, demonstrating increased growth rate of primary follicles. Yet, DHEA also increased oxidative stress, and decreased the number of viable ovarian cells, while increasing early apoptotic cells [62]. The same group later further defined DHEA effects in the mouse ovary [63], demonstrating that, like T, DHEA potentially induces clinically beneficial and detrimental effects on follicle maturation.

These authors also demonstrated distinct immunologic DHEA effects on the ovary: DHEA-induced hyperandrogenism increased T lymphocytes in ovarian tissue, modifying the phenotype by decreasing helper T cells (CD4^+^) and decreasing suppressor/cytotoxic T cell (CD8^+^) [62]. Luchetti et al [45] and Sander et al [46] had previously reported similar immune effects of DHEA, the latter group also demonstrating increases in serum tumor necrosis factor-alpha (TNF-α). CD4^+/^CD8^+ ^cells and TNF-α are, of course, intimately involved with autoimmunity [64,65], a potentially important observation, considering suggested antagonistic activities of androgens in regards to the excessive autoimmune risk of women [32].

Existence of a DHEA-induced animal model for PCOS [45,46,61,62], quite surprisingly, for the longest time did not initiate human investigations of DHEA as an enhancing agent in ovarian stimulation. When Casson et al did investigate DHEA in so-called poor responders [66], they were not motivated by an almost 20 years earlier described rat model [61] but by the observation that DHEA increases IGF-1 [67]. Though they reported mild improvements in oocyte yield after short-term DHEA supplementation [66], they did not further pursue the clinical use of DHEA, failing to recognize its potential clinical importance.

This was left to a series of studies pursued at our center [47,68,69]. Here, correlations to above described mouse model of DHEA were almost startling, when a 43 year old woman with severe DOR, supplemented with DHEA over almost one year (and 9 IVF cycles), developed ovaries with distinctive PCOS phenotype, requiring declining gonadotropin dosages for fear of ovarian hyperstimulation [47].

After self-administering DHEA during sequential IVF cycles, she, since, has been acknowledged as the initiator of worldwide DHEA research [60]. For details on the utilization of DHEA in women with DOR the reader is referred to a recent review, also listing relevant references not included here [60]. Reported data, so far, suggest that DHEA supplementation in women with DOR improves oocyte and embryo yields, oocyte and embryo quality, spontaneous and IVF pregnancy rates and reduces embryo aneuploidy as well as miscarriage rates.

Published DHEA data were initially received with skepticism because all but one small study [70] published so far were neither blinded nor randomized and, therefore, subject to significant potential biases. Larger scale prospectively randomized clinical trials in the U.S. and in Europe had to be abandoned for lack of patient recruitment, as women with severe DOR, understandably, refused randomization [69]. Other clinical trials are still in progress [60].

Viewing published clinical DHEA data with here described animal data in mind, they, however, suddenly, appear more credible: If androgens can beneficially affect primordial follicle recruitment [15,58], why would improving oocyte yields with long-term DHEA supplementation surprise? If androgens can beneficially affect especially small preantral and antral follicles [15-17], why would better egg and embryo quality surprise? Indeed, wouldn't one then also, as has been reported [69], expect increasing DHEA benefits over a few months of supplementation, as broader maturing follicle cohorts get exposed to DHEA?

Furthermore, since small follicles are best assessed by AMH, wouldn't reported improvements in AMH levels after DHEA supplementation [71] be exactly what one expects? And if androgens beneficially affect meiotic maturation [7] and resumption [22], why would improved aneuploidy and miscarriage rates surprise? More on this, though, below.

### Other androgens in human subfertility

Laufer extensively covered until 2007 human androgen experiences in women with DOR [9]. He concluded that differences in patient selection criteria, types of androgens used, varying treatment schedules and the obvious scarcity of studies preclude definite conclusions. Based on data from use of aromatase inhibitors (AI), however, he suggested that promoting an androgen-rich follicle environment may improve IVF outcomes in women who, previously, have failed standard IVF protocols.

But even this minor conclusion needs to be viewed with caution, as documented by Lossl and associates, who in an initial publication reported beneficial effects of aromatase inhibitors on embryo quality [72] but, two years later, in a prospectively randomized study, failed to demonstrate such benefits [73]. Considering earlier noted differences in affinity of androgens to the AR [10], and evidence that the same androgen, at different concentrations, may have different effects [6], contradictory conclusions should, however, not surprise. With T and DHT most agonistic for AR [10], studies utilizing these two hormones would appear of greatest clinical interest. Our literature search, indeed, revealed additional studies of relevance:

Frattarelli and associates also reported contradictory studies: In a first study they suggested that low T levels (< 20 ng/dL) are associated with significantly decreased pregnancy chances in IVF [74]. But two years later they reported only an association between low T levels and IVF stimulation parameters, though no longer with pregnancy chances [75]. Utilizing transdermal T, Massin et al reported no benefits [76], while Balash and associates reported improved ovarian gonadotropin responses in two studies [77,78].

Sipe and Van Voorhis reported improved follicular response of gonadotropin stimulation after T patch administration in a single case of Kallmann's syndrome [79]. The same group, however, reported in a small prospectively randomized, placebo-controlled cross-over study no effects from short-term (12-day) transdermal T supplementation [80].

In contrast, Hossein Rashidi et al noted that T on day 14 after embryo transfer is predictive of IVF pregnancy chances [81], and Qin et al that basal T levels were predictive of number of large follicles on day of ovulation induction, and of pregnancy in women with DOR but not in women with normal ovarian reserve.

Basal T levels were in this study also associated with length of gonadotropin stimulation and total gonadotropin dosage, suggesting that low T appears associated with DOR [82]. In concurrence, Kim et al reported in a prospectively randomized study that transdermal T supplementation by gel resulted in significantly lower total gonadotropin dosages and stimulation length, significantly higher oocyte yields, more mature oocytes, more fertilized oocytes, more good quality embryos, and higher implantation as well as clinical pregnancy rates [83].

Most recently published data, thus, appear supportive of beneficial effects of T on ovarian response in women with DOR. Quin et al with their study offer yet another explanation for contradictory reports in the literature [82]: By demonstrating a possible association between low T levels and DOR, they suggest that primarily patients with low androgen levels may benefit from supplementation. Indeed, absence of treatment effectiveness in women with normal ovarian reserve suggests that androgen supplementation may be most effective in hypoandrogenic women with DOR.

Such conclusions would also be supported by the findings of Hossein Rashidi et al [81] and, ultimately, by earlier noted animal experiments in mice [10,15-18]. If further confirmed, androgen deficiency, like previously for decades androgen excess [84], has to be added as possible cause of female infertility.

### Synergism between androgens and gonadotropins

Various observations support direct synergism between androgens and gonadotropins in the ovary. Li et al recently reviewed this synergism to some degree by formulating a new hypothesis, proposing that during evolution expression of AR progressively shifts from oocytes to theca cells, only to, ultimately, completely disappear in oocytes, eliminating in mammalian oocytes the non-genomic androgen pathway, unlike in somatic cells, thus replacing the function of androgens and ARs in promoting meiotic maturation with gonadotropins [7].

They also note how elusive the role of androgens and AR in the ovary has remained. In earlier studies they had found that only AR in oocytes, though not in somatic cells, can positively affect porcine oocyte maturation [85]. This, however, represents a typical amphibian rather than mammalian characteristic, with the latter, instead, characterized by meiotic promoters like FSH and LH [7]. They, therefore, suggest that driven by evolution, androgens over time have undergone a shift from dominance to cooperation with gonadotropins. As a consequence, in mammals, androgens no longer "control" follicle maturation (a function assumed by gonadotropins) but exert selective control, primarily in earlier stages of follicle maturation.

The authors further suggest that AR in mammalian oocytes may be unnecessary, even harmful, and hypothesize that abundance of AR in granulosa, but also theca, in preantral and antral follicles, previously noted in our discussion of animal data [10,15-17], could prevent excess androgen from entering the oocyte, thus assuring normal follicle growth. Unnecessary androgens under such a concept could, however, still enter the oocyte, bind to remnant AR, triggering non-genomic effects, and forming dysfunctional follicles if abnormal communications exist between granulomas cells and oocyte [7].

As noted before, androgens enhance FSH-driven granulosa cell differentiation and follicle development especially at antral stages [10,29]. Since this developmental stage of the follicle also corresponds to highest AR concentrations in granulosa cells [10,15-17], it is reasonable to assume that, if androgens exert beneficial effects on follicle maturation, this must be the stage of maximal beneficial androgen effects.

One, therefore, can also hypothesize that at this stage external androgen supplementation may most benefit from concomitant external FSH supplementation. Furthermore, assuming a therapeutic range for androgens, as previously discussed, then one also has to assume a "fitting" FSH level to achieve maximal benefits for granulosa cell differentiation and follicle development at this stage. Indeed, one can even further speculate that failure to achieve such an androgen/FSH balance may be detrimental to normal follicle development. A natural balance between androgens and gonadotropins may, indeed, exist, based on the work of Casson et al, who reported that ovarian hyperstimulation augments adrenal dehydroepiandrosterone sulfate secretion [86].

All of these considerations, therefore, suggest the potential of a new therapeutic ovarian stimulation approach in DOR, utilizing this synergism between androgens and FSH, in long-term parallel supplementation of granulosa cells with androgen as well as FSH (Figure 1). The first patient supplemented with DHEA at our center, indeed, underwent nine consecutive gonadotropin-stimulated IVF cycles while on DHEA supplementation [47]. She has remained one of the most responsive patients to DHEA we have seen in over seven years, was one with the longest concomitant FSH supplementation, and, thus, amongst the longest patients exposed to the potential benefits of such synergism.

Patients undergoing a large number of consecutive cycles are rare. We, however, have had the opportunity to follow a small number of such patients. Our preliminary impression is that, especially in women with very severe DOR, we see better oocyte yields and quality with consecutive ovarian stimulation cycles, which allow for a synergistic androgen/FSH effect (Gleicher N, Barad DH, unpublished data). A more formal study is underway.

If confirmed, one then also could expect better cumulative pregnancy rates. This then would be potentially support a radically new ovarian stimulation protocol in women with DOR, utilizing, in combination, long- term androgen and low dose gonadotropin support, after a few weeks to months intermittently followed by high dose gonadotropin stimulation episodes, potentially resulting in egg retrieval. This kind of synergism between androgens and FSH is also strongly supported by animal data [87].

### Arising concepts

Our DHEA supplementation experience in women with very severe DOR (undetectable AMH to 0.3 ng/mL), based on surprisingly good pregnancy and live birth numbers [88,89], unexpectedly low miscarriage rates [90] and reduced embryo aneuploidy [91], led us to conclude that existing concepts of ovarian aging require reconsideration. Aging oocytes, simply, no longer appear logical. We, instead, suggested that the unrecruited oocyte at primordial follicle stage does not age. Instead, it is the ovarian environments, where follicle maturation takes place after recruitment that age. Consequently, the poorer environment of older ovaries negatively affects the maturation process of, initially perfectly healthy, oocytes [92].

Here presented review of androgens is supportive of such a modified concept of ovarian aging. The review demonstrates that androgens, in contrast to long held beliefs, are not always detrimental to follicle maturation, and, within therapeutic ranges, indeed, can be clinically beneficial. Published data on the subject have for a variety of reasons been inconsistent (Table 2). As here presented, it is apparent that follicles are subject to androgen effects from the earliest maturation stages on, likely including recruitment, reaching peak intensity at preantral and antral stages, androgens primarily affect granulosa cells, inducing ligand-activated AR, which in turn modulates FSH action in developing granulosa cells, modulating differentiation and, consequently, follicle development (10 and Figure 1).

**Table 2:** Reasons why study results on androgen effects have, likely, been inconsistent

Reasons why study results on androgen effects have likely been inconsistent
Not all androgens are equally agonistic with AR
Androgens have genomic and non-genomic effects on follicle maturation
A specific androgen may have different effects:
• Based on concentration: inside or outside of therapeutic ranges
• Based on synergism/antagonism with other factors: synergism with FSH, etc.
Androgens affect follicles differently at different maturity stages:
• Most effective at preantral and antral stage
• Length of androgen supplementation, therefore, counts
Androgens may be only/most effective in hypoandrogenic/DOR patients
Inhomogeneous study populations: PCOS as example

As ovaries age, it is reasonable to assume that, like in other organs, tissue processes decline in efficacy. For example, DHEA levels significantly decrease with advancing age [93], probably mostly the result of changes in adrenal function. In contrast, T and free androgen index increase but sex hormone-binding globulin decreases [94], while FSH, of course, increases [95]. It, therefore, appears very likely that androgenic follicle environments, in which oocytes mature, change as women age. And so do, of course, as a consequence, androgen/FSH ratios, as previously noted synergistically acting on granulosa cells (Figure 1).

These are likely, only two amongst many more, adverse ovarian consequences due to older age. They, however, at least partially can be reversed via androgen supplementation, whether in form of DHEA, T or of other androgens. The importance of normal androgen levels, likely in a certain ratio to FSH levels, especially at preantral and antral follicle stages, therefore, appears obvious.

We noted before that based on varying degrees of agonism with AR, different androgens exert varying effects [10]. They, however, also affect ovarian processes in non-genomic ways, adding additional potential variability [10,13]. For example, we recently reported that more androgenic progestational agents in oral contraceptives suppress ovarian reserve more severely than less androgenic compounds [96], reemphasizing not only the variability of androgenic ovarian effects but their, at times, outright opposing effects. Some improve [71], and others suppress FOR [96].

Clinical research over the last half century almost exclusively concentrated on the last two weeks of follicle maturation, the gonadotropin-sensitive phase of folliculogenesis. Here reviewed from a clinical vantage point, androgen effects on follicle maturation may not only be relevant for DOR patients but may offer the far larger potential of a revolutionary new approach towards therapeutic follicle maturation at much earlier stages of folliculogenesis.

Such early therapeutic interventions into follicle maturation will, however, only be successful if, as we suggested [92], oocytes do not age as long as unrecruited. Oocyte aging at primordial stage, traditionally held responsible for declining fecundity and increasing aneuploidy with advancing female age [97,98], would render subsequent therapeutic interventions futile since already damaged oocytes, even with treatment, cannot be expected to recover [92].

Only assumption of undamaged, perpetually "young" oocytes at time of recruitment allows for successful therapeutic interventions post-recruitment, improving the ovarian environment and, therefore, the quality of oocyte maturation, in turn leading to better oocyte and embryo quality.

Such a concept is not entirely new: Hodges et al suggested almost 10 years ago that treatments with potential to reduce age-associated aneuploidy by beneficially influencing meiotic chromosome segregation may become possible. They consider congression failure (disturbances in chromosome alignment of meiotic spindles) responsible for aneuploidy, and believe that a variety of signals regulating folliculogenesis, together with increased risk of non-disjunction errors, can be therapeutically affected [99].

Androgens may be a first class of agents that do exactly that! After all, androgens (in combination with FSH) stimulate resumption of meiosis in ovaries and testes, while AR gene inactivation blocks the effect [7,10,19,22,100].

Romero and Smitz demonstrated that, at certain concentrations, ASD-treated follicles *in vitro *produce oocytes with chromosome displacements on metaphase plate [6]. Androgens, therefore, may very well affect ploidy in oocytes. Some, like DHEA in humans, may reduce aneuploidy [91] and others may, potentially, exert opposite effects.

But not only androgens may beneficially affect early stages of follicle maturation. Since in practically all tissues mitochondrial functions are lost with advancing age, Bentov et al proposed in women with DOR treatment with mitochondrial nutrients [101].

In a mouse model such treatment proved effective [102]. Treatment of DOR with androgens may, therefore, only a first step in improving the ovarian environment in which follicles mature.

## Abbreviations

AI: Aromatase inhibitor; AFC: antral follicle count; AMH: anti-Müllerian hormone; AR: androgen receptor; ARKO: androgen receptor knock out; ASD: Androstenedione; DHEA: dehydroepiandrosterone; DHT: dehydrotestosterone; DOR: diminished ovarian reserve; FOR: functional ovarian reserve; FSH: follicle stimulating hormone; hCG: human chorionic gonadotropin; IVF: in vitro fertilization; LH: luteinizing hormone; mRNA: messenger ribonucleic acid; PCOS: polycystic ovary syndrome; POF: premature ovarian failure; SHBG: sex hormone-binding globulin; T: testosterone; TNF: tumor necrosis factor

## Competing interests

All three authors have in the past received research support, speakers' honoraria and travel funds from various pharmaceutical and medical device companies, none, however, related to the subject of this paper. N.G. and D.H.B. are listed as co-inventors of an awarded U.S. patent, claiming therapeutic benefits for DHEA, and potentially other androgens, in women with diminished ovarian reserve. Both authors have other patent applications, regarding DHEA, and potentially other androgens, and, unrelated to this presentation, the *FMR1 *gene's effects on ovaries, pending.

## Authors' contributions

NG developed the concept for the study, reviewed all publication included in the review of the literature and was the principal author of the manuscript. DHB reviewed selected publications, contributed to design of the study, was responsible for statistical considerations of the review and contributed to the writing of the manuscript. AW contributed to the concept of the study, and contributed to the writing of the manuscript. All three authors approved the final version of the manuscript.
